# Phenolic Composition and Antioxidant Activity of Peel, Pulp and Seed Extracts of Different Clones of the Turkish Grape Cultivar ‘Karaerik’

**DOI:** 10.3390/plants10102154

**Published:** 2021-10-11

**Authors:** Muhammed Kupe, Neva Karatas, Mehmet Settar Unal, Sezai Ercisli, Mojmir Baron, Jiri Sochor

**Affiliations:** 1 Department of Horticulture, Faculty of Agriculture, Atatürk University, Erzurum 25240, Turkey; muhammed.kupe@atauni.edu.tr; 2 Department of Nutrition and Dietetics, Faculty of Health Sciences, Ataturk University, Erzurum 25240, Turkey; ngungor@atauni.edu.tr; 3 Department of Horticulture, Faculty of Agriculture, Sirnak University, Sirnak 73000, Turkey; munal62@hotmail.com; 4 Department of Viticulture and Enology, Faculty of Horticulture, Mendel University in Brno, Valticka 337, 691 44 Lednice, Czech Republic; mojmirbaron@seznam.cz (M.B.); jiri.sochor@mendelu.cz (J.S.)

**Keywords:** grape, antioxidant activity, peel, pulp, seed

## Abstract

The Erzincan plain is one of the richest regions in Turkey in terms of plant biodiversity. In this region, the famous grape cultivar ‘Karaerik’ has always dominated grape production due to its berry characteristics. The cultivar shows great morphological variation at clonal level. In this study, the total phenolic content and antioxidant activity of peel, pulp and seed extracts of nine ‘Karaerik’ clones sampled from same location were investigated. The Folin–Ciocalteu method was used to determine the total phenolic content of peel, pulp and seed extracts of nine clones. To determine antioxidant activity, three well known assays such as DPPH (2,2-diphenyl-1-picryl-hydrazyl-hydrate), FRAP (Ferric Reducing Antioxidant Power) and TEAC (Trolox Equivalent Antioxidant Capacity) were used. In addition, the correlation between total phenol content and DPPH, FRAP and TEAC was determined. Results showed that among the tissues, seed samples in berries of all clones had the highest total phenol content and antioxidant activity determined by three assays. Seed samples were followed by peel and pulp for total phenolic content and antioxidant activity. Among the nine ‘Karaerik’ clones, Clone 8 had the highest total phenolic content (149 mg GAE/100 g FW) while Clone 3 had the lowest (111 mg GAE/100 g FW). Peel, pulp and seed samples of nine ‘Karaerik’ clones showed strong antioxidant activity in DPPH, FRAP and TEAC assays. In particular, grape seeds were found rich for better in phenolic compounds including gallic acid, quercetin, catechin, chlorogenic acid, caffeic acid and *p*-coumaric acid. Clones such as 7, 8 and 9 higher antioxidant activity may present great potential for grape breeders and the food industry as well as health-conscious consumers.

## 1. Introduction

Turkey has a superior geographical and ecological advantage in terms of the cultivation of horticultural plants including fruits, vegetables and grapes. Due to its different climatic conditions, many fruit and vegetable species and two main *Vitis* species (*Vitis vinifera* and *Vitis labrusca*) have been grown in different regions of the country since ancient times. Located in both the Near East and the Mediterranean basins, Turkey is the place of the genetic origin of many horticultural species. This situation becomes even more advantageous when combined with the advantage of ecology. As a matter of fact, out of 138 fruit and 100 vegetable species cultivated in the world today, more than 80 fruit species and 50 vegetable species can be grown in Turkey [1,2,3,4,5].

It is strongly recommended to consume horticultural crops including grape berries daily due to their high phenolic compounds, which have beneficial effects on human health. Phenolic compounds, widely found in particular horticultural crops, are secondary metabolites produced in the shikimic acid of plants and pentose phosphate through phenylpropanoid metabolization [6]. They include benzene rings and form simple form phenolic to highly polymerized compounds [7]. Phenolic substances or polyphenols are distributed throughout the entire metabolic process in plants and contain numerous compounds such as flavonoids, phenolic acids, anthocyanins, etc. [8]. Horticultural crops are the main sources of phenolic compounds in the human diet compared to other crops. Plant polyphenols as natural and dietary antioxidants in human health and disease might offer some protection against oxidative damage [9]. The majority of horticultural crops especially, grapes, small fruits and berries are rich in phenolic compounds, for example phenolic acids and flavonoids, and promote health benefits by reducing the risk of metabolic syndrome and related complications such as type 2 diabetes [10]. Moreover, the antioxidant compounds found in horticultural crops are mainly phenolic and include compounds such as tocopherols, carotenoids, phenolic acids (benzoic acid derivatives and cinnamon acids), flavonoids, and dipropenes. Secondary plant-derived metabolites, including phenolic compounds, have a potent potential to clear free radicals that exist in all parts of the plant, such as the leaves, fruits, seeds, roots, and skin [11]. Studies have shown that many factors, such as climatic, geographic, genetic, extraction methods, etc., affect the amount of secondary metabolites in plants [12,13].

Grape (*Vitis vinifera*) berries are rich in phytochemicals and these substances may be important for reducing chronic illnesses, such as cancer, cardiovascular diseases, ischemic stroke, neurodegenerative disorders and aging [14,15,16,17]. Grapes are one of the richest sources of phenolic substances and antioxidant compounds among fruits. Therefore, grape berries have been broadly studied due to their composition in phenolic substances and antioxidant compounds and their potential beneficial effects on human health. Studies have shown that grape cultivars have differences in terms of phenolic substances and antioxidant capacity. Moreover, different plant parts such as leaves, seeds, berry peel, and berry pulp show differences in terms of antioxidant activity. Among grapes, black varieties and their products contain rich nutritional and phenolic content [18,19,20,21,22,23].

Along with cultivars and plant parts, many other factors such as environment, viticultural practice, rootstocks, etc., affect phenolic compounds and the antioxidant activity of grape berries [24,25,26,27]. In addition, during the long cultivation periods of grapevines, mutations occur in vineyards and grape varieties generate populations of individuals that may present certain differences. This intra-varietal diversity is expressed in many ways: growth habit, vigour, fertility, phenological cycle, accumulation of sugars, aromatic and/or polyphenolic potential, size and shape of bunches and berries, compactness of bunches, ampelographic characteristics (color of organs and indentation of the leaves), etc. Thus, clonal variation is common in grape cultivars and different clones of same cultivar affect their antioxidant activity and have an influence on the antioxidant activity [28].

The Erzincan plain, located in eastern Anatolia, has a long tradition of viticulture. ‘Karaerik’ cultivar is the most important black grape variety grown in Erzincan and is used for the production of high-quality table grapes. The cultivar has large, attractive black berries with a perfect sugar–acid balance. ‘Karaerik’ means black plum in English. The origin of this cultivar is unknown and shows low adaptation capacity; thus, they are not successfully introduced in the other grape-growing regions with different climatic conditions in Turkey. The plantations of this cultivar in Erzincan have a heterogeneous population [29,30]. Using the complex selection criteria, it is possible to make a better clonal selection from this cultivar [31]. In the Erzincan plain, the ‘Karaerik’ cultivar exhibits different clones that vary in their berry biochemical properties [32]. Clonal selection is considered a very important tool for grapevine genetic improvement. In most of the grapevine-growing regions of the world, selection has always been a successful breeding method. Clonal selection is a means of intensive improvement successfully adapted in several varieties such as White Riesling, Muscat d’Adda, Pinot sp., Steinschiller, Hárslevelû, Welsch/Italian Riesling, Müller Thurgau, etc.

The aim of this study was to evaluate the phenolic compounds and antioxidant activity in peel, pulp and seeds of nine ‘Karaerik’ clones in order to find differences and/or similarities between ‘Karaerik’ clones.

## 2. Materials and Methods

### 2.1. Plant Materials, Sampled Vineyards and Location

Nine ‘Karaerik’ clones were harvested from different vineyards in the Uzumlu district of Erzincan in the 2020 growing season. The sampled vineyards are around 15 years old with a specific ‘Baran’ training system. The berries were picked homogenously and their laboratory analyses were conducted after the morphological measurements. 

### 2.2. Morphological Traits

Harvest period, number of seed, cluster weight, cluster form, berry weight and berry color were performed in the full ripening period, in a sample of 3 kg of clusters. Characterization of the berries (weight and peel color) was performed in a representative sample of 40 berries taken randomly form the middle part of clusters [33]. Analysis was performed in duplicate.

### 2.3. Extraction

The fresh grape pulp, peel and seed extracts were obtained by [34]. The extraction sample (2 g) was put into centrifuge tube and then 8 ml acidic methanol–water (60:40, *v/v* pH 2) was added. The centrifuge tube was vortexed for 1 min and shaken for 1 h. After that, the sample was centrifuged at 9000 rpm/10 min at 4 °C to recover the supernatant. A total of 8 mL of acetone–water (70:30) was added to the residue before it was stirred and sonicated for 1 and 15 min, respectively, and centrifuged again at 4 °C and 9000 rpm/10 min. The last extraction was repeated without sonication. The obtained supernatants transferred to a 25 mL volumetric flask, and acetone–water (70:30) was added to reach a final volume of 25 mL. Finally, the extract was stored at −80 °C until analysis. 

### 2.4. Total Phenol Content

The Folin–Ciocalteau method [35] was used to determine the total phenolic content in peel, pulp and seed samples of cv. ‘Karaerik’ grape clones. To achieve this, 9 mL of 80% methanol was added to 1 mL of the sample and the mixture was centrifuged at 5500 rpm for 10 min. After that, 50 µL of supernatant was added to 250 µL of the Folin–Ciocalteu reagent. Then, 750 µL 20% (w/v) Na_2_CO_3_ was supplemented and incubated for 2 h. Afterward, the absorbance was measured at 760 nm against a blank surface. The results are expressed as milligrams of GAE (gallic acid equivalent) per 100 g of fresh weight (FW). Analysis was performed in duplicate.

### 2.5. Total Antioxidant Capacity Measurement

#### 2.5.1. 1,1-. Diphenyl-2-picryl-hydrazyl Assay 

Brand-Williams et al.’s [36] method was used to determine the 1,1-Diphenyl-2-picryl-hydrazyl (DPPH) scavenging capacity of peel, pulp and seed parts in berries of cv. ‘Karaerik’ clones. First, DPPH powder was dissolved in methanol to obtain an absorbance of 0.7 ± 0.02 at 517 nm. An amount of 20 μL test sample was diluted with water, Trolox standard or blank (distilled water) and was placed in a 96-well microplate, after which 280 μL of working DPPH solution was added. A total of 30 min later at 30 °C, the absorbance was measured at 517 nm using a microplate reader. Aqueous solutions of Trolox (50–500 μM) were used for calibration. Finally, the obtained results were expressed micromoles of Trolox equivalent (TE) per 100 g of fresh weight (μmol of TE/100 g of FW). 

#### 2.5.2. Ferric Reducing Antioxidant Power 

Benzie and Strain’s [37] method was used for ferric reducing antioxidant power (FRAP) determination. The amount of 20 μL of the test sample, diluted with water, or Trolox standard, or ferrous sulphate standard or blank (distilled water) was placed in a 96-well microplate, after which 280 μL of the FRAP reagent (containing TPTZ, FeCl3 and acetate buffer), freshly prepared and warmed at 37 °C, was added. After 30 min, the absorbance at 595 nm was measured. The standard curves were constructed using FeSO4 (115–1150 μM) and Trolox solutions (20–400 μM) and the results were expressed as micromoles of Trolox equivalent (TE) per 100 g of fresh weight (μmol of TE/100 g of FW).

#### 2.5.3. Trolox Equivalent Antioxidant Capacity 

Re et al.’s [38] method was used for Trolox equivalent antioxidant capacity (TEAC) determination. To obtain ABTS stock solution, 7 mM ABTS and 2.45 mM potassium persulphate in a volume ratio 1:1 was prepared and, after that, incubated in the dark for 16 h. ABTS stock solution was diluted with phosphate buffer 5 mM at pH = 7.4 to obtain an absorbance of 0.7 ± 0.02 at 730 nm. An amount of 30 μL of the test sample, diluted with water, or Trolox standard or blank (distilled water) was placed in a 96-well microplate, after which 270 μL of radical ABTS was added. A total of 30 min later, the absorbance was measured at 730 nm. Aqueous solutions of Trolox concentrations (20–200 μM) were used for calibration and the results were expressed as micromoles of Trolox equivalent (TE) per 100 g of fresh weight (μmol of TE/100 g of FW). 

### 2.6. Phenolic Compounds 

The phenolic compounds in grape samples were determined following the procedure described by Rodriguez-Delgado et al. [39]. About 50 g sample for each sample was transferred to a centrifuge tube, mixed homogeneously, then diluted 1:1 with distilled water and centrifuged at 15,000× *g* for 15 min. The supernatant was passed through a 0.45 µm Millex-HV Hydrophilic PVDF membrane filter, then injected into the HPLC system (gradient). The chromatographic separation in Agilent 1100 series HPLC took place in a DAD (photodiode array detector) detector (Agilent, Waldbronn, Germany) with 250 mm × 4.6 mm, 4m ODS column (HiChrom, New Jersey, USA). The following solvents in water with a flow rate of 1 mL/min and 20 µl injection volume were used for spectral measurements taken at both 254 nm and 280 nm: as mobile phase solvent A, methanol–acetic acid–water (10:2:88) and Solvent B, methanol–acetic acid–water (90:2:8). The results were expressed as mg/100 g FW.

### 2.7. Statistical Analysis 

The study was planned as four replication including 10 samples per replicate. In the statistical evaluations, Windows SPSS 20 (IBM Corp. Armonk, NY, USA) was used and the differences between the means was evaluated by subjecting to ANOVA variance analysis and determined with Duncan multiple comparison test (*p* < 0.05). The Pearson correlation analysis between antioxidant activity and total phenolic content was performed. 

## 3. Results and Discussion

### 3.1. Morphological Traits

Table 1 presents some important morphological traits of the nine clones of ‘Karaerik’ grape cultivar. The clones exhibited diversity for most of the searched morphological parameters. 

In terms of harvest season, Clones 1, 7 and 9 were found earlier than the rest of the Clones. (Table 1). Clones 9 had the lowest number seeds per berry, Clones 1, 4, 6, 7 and 8 had 2-3 seeds per berry and Clones 2 and 3 had the highest average of the seeds per berry (3-4) which reflects to the larger size of the berries obtained (6.13 g for Clone 2 and 6.90 g for Clone 3). 

Clone 9 gave the lowest berry weight (4.59 g, which had the lowest number of seeds) (Table 1). All clones had a black berry color and all of them were to be used only for table consumption due to their perfect fresh berry characteristics (larger size, attractive color, unique sugar-acid balance, thick peel). 

**Figure 1:**
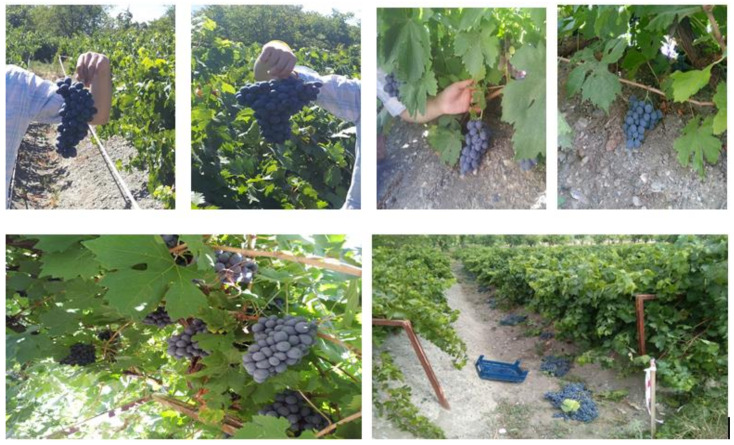
‘Karaerik’ grape cultivars (in ‘Baran’ training system).

Cluster form was found to be different among Clones (Figure 1), with Clones 1, 2 and 3 having a winged cylindrical form, Clone 4 having a winged conical form, Clones 5 and 6 having an irregular winged conical form and the rest of the Clones having a conical cluster form (Table 1). The average cluster weight of the Clones was found to be between 346 and 587g, with the highest cluster weight obtained from Clone 3. 

According to OIV [33], cluster weight of all Clones was found to be over 300 g and classified as large. In grapes, cluster and berry weight are strongly affected by cultivar, altitude, cluster thinning, etc. Kok et al. [22] reported cluster weight in grape cultivars between 232 and 560 g, which indicated good agreement with our result. Dilli and Kader [40] studied table grape cultivars widely grown in Turkey and reported that the cultivars were harvested early, mid and late period. They also found that common table cultivars had a large cluster weight and 1–4 seeds per berry. Good quality in table grapes represents a combination of medium-sized clusters of uniformly large, perfect berries with the characteristic color, pleasing flavor, and the texture of the cultivar. Uniform color formation and suitability for transportation are also desirable traits for table grapes [41].

Our results are in agreement with the above studies [22,39]. The differences could be effects of the different cultivars, altitude, ecology, etc. In the literature, there were studies that determined the morphological characteristics of grape cultivars, indicating a wide variability in cluster weight, cluster form, seed number per berry, berry color and berry weight, according to cultivars and treatments [42,43,44]. We found great diversity in particular cluster forms even in the same vineyards.

### 3.2. Total Phenolic Content

The results of the total phenolic content of peel, pulp and seed samples of nine ‘Karaerik’ grape clones are presented in Table 2. The Clones showed statistically significant differences each other (*p* < 0.05) in terms of peel, pulp and seed total phenolic content (Table 2). 

Among the nine ‘Karaerik’ clones, Clone 8 had the highest total phenolic content in peel as 149 mg GAE/100 g FW, and followed by in descending order Clone 9 (143 mg GAE/100 g FW) > Clone 5 (138 mg GAE/100 g FW) > Clone 7 (134 mg GAE/100 g FW) > Clone 2 (129 mg GAE/100 g FW) > Clone 6 (126 mg GAE/100 g FW) > Clone 4 (121 mg GAE/100 g FW) > Clone 1 (117 mg GAE/100 g FW) > Clone 3 (111 mg GAE/100 g FW), respectively (Table 2).

For pulp, the Clones exhibited the lowest total phenolic content, which varied from 2.775 to 3.715 mg GAE/100 g FW (Table 2).

The seed of all Clones displayed the highest total phenolic content. The differences total phenolic content in seeds of nine Clones was found to be significant at *p* < 0.05 level. Clone 6 had the highest total phenolic content at 245 mg GAE/100 g, followed by Clone 9 at 238 mg GAE/100 g FW, whereas the lowest total phenolic content was observed in Clone 1 and Clone 4 at 201 and 207 mg GAE/100 g FW, respectively (Table 2). 

The results clearly indicated that grape seeds are richer sources of total phenolic content than peel and pulp and also peel was found to be richer than pulp for all nine Karaerik grape clones. It is also evident that there are clonal variations among grape clones in terms of total phenolic content (Table 2). In fact, there were a few studies comparing the total phenolic content of peel, pulp and seed samples of different grape cultivars and even a very limited number of studies has been carried out on different clones of a single cultivar. Yi et al. [45] observed great variability in juices among grape cultivars in terms of total phenolic content in the range of 44–184 mg GAE/100 g fresh samples, which is in accordance with our results. In Spain, Ruiz-Torralba et al. [46] used berries of two white and red grape cultivars and reported total phenolic content values between 124 and 151 mg GAE/g FW. A large number of grape cultivars were used in total phenol content analysis in Italy and great variation has been observed among cultivars in terms of total phenolic content (92–468 mg GAE/100 g FW [47]. In middle Anatolia, Gundesli et al. [48] determined the average total phenol content of 222 mg GAE/g FW in berries of the Kabarcik grape cultivar. In China, Liu et al. [49] used a large number of diverse grape cultivars including white, red and black colored cultivars and found total phenol content varied from 29 to 140 mg GAE/100 g FW. In China, a total six red peel colored grape cultivars was used in an experiment and the total phenolic content was found to be the highest in seeds, followed by peels and pulps, indicating similarities with our study [50]. Yilmaz et al. [51] produced a comprehensive study to determine the total phenolic content and antioxidant activity of 22 grape cultivars including seven white and 15 red grapes grown in the Marmara region of Turkey. They found that total phenolic contents of grape pulp, seed and peel parts ranged from 9.26 to 62.29, from 162.29 to 326.18 and from 96.61 to 167.42 mg gallic acid equivalents/100 g fresh weight among cultivars, respectively. Our finding is coincided with the results of Yilmaz et al. [51]. Previous studies indicated that total phenolic content vary among plant organs of grape, cultivars, clones, growing location, climate, soil, temperature, cultural practices, ripening stage, training system, etc. [51,52,53,54,55]. Phenolic compounds contribute the color and taste characteristics of grapes and they also significantly contribute to the antiradical and antioxidant properties of grape berries [56]. The contributions of grape juice, pulp, peel and seeds to the total phenolic contents of grape berries were reported to be 5, 1, 30 and 64%, respectively [57]. Karaman et al. [58] reported that total phenolic content cultivar dependent and seeds were found to be richer than peels of all six grape cultivars used. 

### 3.3. Total Antioxidant Capacity

#### 3.3.1. DPPH Assay

DPPH (1,1-diphenyl-2-picrylhydrazyl) analysis is one of the best-known, accurate, and frequently employed methods for evaluating antioxidant activity in plant materials [59]. The DPPH scavenging against ‘Karaerik’ grape peel, pulp and seed extracts was found in the descending order of seed>peel>pulp for all nine Clones (Table 3). There were statistically significant differences among Clones for DPPH scavenging activity (*p* < 0.05). The highest DPPH radical scavenging were observed in Clone 9 as 1918 μmol Trolox/100 g FW while the lowest values were obtained from Clone 1 as 1510 μmol Trolox/100 g FW. The peel samples of nine ‘Karaerik’ grape clones exhibited DPPH radical scavenging between 1080–1340 μmol Trolox/100 g FW. The lowest DPPH scavenging has been observed in pulp samples were in range of 276–346 μmol Trolox/100 g FW, respectively (Table 3). It is clear that ‘Karaerik’ grape seeds and peels extracts belongs to different clones significantly (*p* < 0.05) inhibited DPPH free radicals’ generation. The studies conducted in different parts of the world on grape peel and pulp. For example, Choi et al. [60] reported higher DPPH radical scavenging activity for Campbell Early grape seed extracts than pulp in Korea. Fahmi et al. [61] used DPPH assay to estimate antioxidant activity in grapes and found that different grape cultivar extracts exhibited variable DPPH radical scavenging effect. Farhadi et al. [62] used six grape seeds and pulps for antioxidant capacity analysis and found DPPH inhibition was the highest in the seed extracts. Mandić et al. [63] reported that grape species, individual cultivars, cultivation condition, maturation stage, and seasonal variations may affect phenolic biosynthesis and antioxidant capacity in grape seed, peel and flesh. Yilmaz et al. [51] indicated that the seeds of grape cultivars are the best source of antioxidants, followed by peels and then pulps by using DPPH, FRAP and TEAC assays. In a study, Anastasiadi et al. [64] reported that regardless of the assay method, grape seeds have the best antioxidant activity compared to peel and pulp. Shen et al. [50] observed that DPPH scavenging effects of the pulp and peel of grape cultivars were remarkably lower than those of the seeds.

#### 3.3.2. FRAP Assay

The antioxidant activities determined by FRAP assay in peel, pulp and seeds of nine Clones were analyzed and expressed μmol Trolox/100 g FW. The Clones differed each other significantly (*p* < 0.05) in terms of FRAP values (Table 4).

As shown in Table 4, the highest FRAP values were expressed from seeds and followed by peel and pulp. The seed extract from Clone 8 had the highest FRAP value (52460 μmol Trolox/100 g FW), whilst that of Clone 9 and Clone 7 seeds showed the second and third highest activity (50640 and 49300 μmol Trolox/100 g FW, respectively). In the FRAP assay, among the seed samples, the lowest activity was observed in Clone 3 as 39880 μmol Trolox/100 g FW. The FRAP values of peel and pulp samples of nine ‘Karaerik’ grape clones varied from 3544 (Clone 3) to 4610 (Clone 9) μmol Trolox/100 g FW and 77 (Clone 3) to 128 (Clone 8) μmol Trolox/100 g FW. Overall, the peel, pulp and seeds of Clone 3 exhibited the lowest FRAP value (Table 4). Liu et al. [49] used pulp samples of 30 common grape cultivars with white, red and black peel color in China and found FRAP values in the range of 59–612 μmol Trolox/100 g FW. Our FRAP results showed some similarities with this study. 

In another study, Fu et al. [65] used whole berry samples of four grape cultivars and found FRAP values between 173 and 1012 μmol Fe (II)/100 g FW. In another study [66] showed that FRAP values of 56 wild edible fruits quite variable and ranged from 67 to 14300 μmol Fe (II)/100 g FW. Sochorova et al. [67] showed that grape seeds had high content of antioxidant components and the differences in content mainly belongs to individual cultivars. Shen et al. [50] used six grape cultivars in FRAP analysis and found that the seeds had higher FRAP values than pulp. Ruiz-Torralba et al. [46] used whole berries of white and red grape cultivars and reported FRAP values between 738 and 786 μmol Trolox/100 g FW, which is in agreement with our present results. Yilmaz et al. [51] used a large number of grape seed, pulp and peel extracts and found that FRAP values were the highest in seed samples. They reported that the pulps of red cultivars had higher FRAP values than white cultivars. Among the red cultivars, the pulps of Hamburg Misketi had an FRAP value of 297 μmol TE/100 g FW. Our results were in good agreement with this report. Most of the studies showed that grape seed contains higher FRAP values than peels and also that peel has higher FRAP values than pulps [50,51,58,68]. Gokturk Baydar et al. [69] demonstrated that seeds of Narince grape cultivar had the highest antioxidant activity than pulp. 

#### 3.3.3. TEAC Assay

The antioxidant activity of peel, pulp and seeds of nine ‘Karaerik’ grape clones was also performed using the Trolox equivalent antioxidant capacity (TEAC) assay. Results of TEAC assay in peel, pulp and seed extracts of nine ‘Karaerik’ grape clones are presented in Table 5. Statistically significant differences among cultivars for peel, pulp and seed extracts are found (*p* < 0.05). For each studied clone, the highest TEAC values was observed in seeds and followed by peel, and the lowest TEAC values are evident in pulp samples (Table 5). For seed extracts, The TEAC values were found between 1464 (Clone 3) and 1730 (Clone 9) μmol Trolox/100 g FW. For peel and pulp extracts, those values were within in range of 310 (Clone 3) to 414 (Clone 9) μmol Trolox/100 g FW and 50 (Clone 3) to 98 (Clone 7) μmol Trolox/100 g FW, respectively (Table 5). Overall, the lowest TEAC values of peel, pulp and seed extracts was observed from ‘Karaerik’ Clone 3. TEAC results clearly indicated that there was a great variability among ‘Karaerik’ clones in terms of TEAC values and results also showed that grape seeds are better sources of antioxidants among grape plant parts even among horticultural crops. Costa et al. [70] also found that grape berries are rich for antioxidants by using the TEAC method. Ruiz-Torralba et al. [46] used whole berries of white and red grape cultivars and reported FRAP values 971 μmol TE/100 g FW for red and 1097 μmol TE/100 g FW for white cultivars. Liu et al. [49] used 30 grape cultivars grown in China and determined the TEAC values of whole berries in the range of 339–4839 μmol TE/100 g FW, respectively indicating similarities with our study. Sochorova et al. [67] indicated that the grape seed extract was rich both in oligomeric and polymeric flavanols; therefore, extracts from grape seeds and peels contained great amounts of antioxidants. Weidner et al. [71] studied phenolic compounds isolated from seeds of European and Japanese species of grapevine (*Vitis vinifera* and *Vitis coignetiae*) and found that seeds contained great amounts of tannins and detectable levels of catechins and *p*-coumaric, ferulic and caffeic acids that have antioxidant effect. Yilmaz et al. [51] used a large number of grape cultivars with different berry peel color and found great variability both among cultivars and plant parts (peel, pulp and seeds) in terms of antioxidant activity by using TEAC assay. They also indicated that DPPH, FRAP and TEAC assays were useful to determine antioxidant activity of different plant parts of grapes. 

#### 3.3.4. Correlations between TPC and Antioxidant Activity

The correlations between the total phenol content (TPC) with antioxidant activity (DPPH, FRAP and TEAC) in peel, pulp and seeds of nine clones of ‘Karaerik’ grape cultivar are shown in Table 6. 

Considering the correlation coefficient, the TPC of grape peel, pulp and seeds showed a high correlation coefficient (0.8 < r < 1) with DPPH (0.84, 0.82 and 0.87), FRAP (0.81, 0.75 and 0.80) and TEAC (0.85, 0.82 and 0.80). DPPH showed a moderate positive correlation (0.5 < r < 0.8) with FRAP (0.72, 0.68 and 0.70) and TEAC (0.55, 0.50 and 0.52) based on peel, pulp and seed. In addition, FRAP also showed a moderate positive correlation (0.5 < r < 0.8) with TEAC (0.73, 0.65 and 0.67) (Table 6). This correlation helps us to understand the contribution of the total phenol content to the antioxidant capacity of grape pulp, peel and seeds in findings reported previously [72,73,74]. Clarke et al. [75] found that high correlation of DPPH, FRAP, TEAC and TPC indicates redundancy in the use of all three assays to screen for the antioxidant activity in extracts of plants.

### 3.4. Phenolic Compounds

It has been observed that phenolic compounds show a wide variation both in berry parts and clones of ‘Karaerik’ grape cultivar (Table 7, Table 8 and Table 9). Phenolic compounds, except ferulic acid, chlorogenic acid, quercetin, myricetin and vanillic acid in the peel of ‘Karaerik’ clones, showed significant differences at p ≤ 0.05 level (Table 7). All ‘Karaerik’ clones had the highest amount of syringic acid (51.1–73.6 mg/100 g FW) in its peel, and followed by caffeic acid (22.0–35.7mg/100 g FW) and gallic acid (10.4–19.1 mg/100 g FW), respectively. 

It was determined that all ‘Karaerik’ clones had negligible amounts of phenolic compounds in pulp. Moreover, all phenolic compounds determined in pulp of nine ‘Karaerik’ grape clones were found insignificant (Table 8).

In the experiment, the seeds of all clones exhibited higher phenolic compound than peel and pulp samples. Gallic acid content in seeds of the examined nine clones generally had higher than the other phenolics. The highest gallic acid content was obtained from Clone 5 as 110.1 mg/100 g FW, while the lowest gallic acid content was determined to be in the Clones 8 and 4 as 95.0 and 95.6 mg/100 g FW, respectively. Next to gallic acid, quercetin was found to be the second most important phenolic compound in ‘Karaerik’ grape seeds, and its concentration varied from 57.5 to 72.1 mg/100 g FW, respectively (Table 9). Pantelic et al. [76] reported that grape seeds rich for phenolic compounds including gallic acid, syringic acid, quercetin, caffeic acid, chlorogenic acid and seeds followed by peel and pulp in terms of phenolic compounds which is good agreement with our results. Rusjan et al. [77] also indicated that grape berries are rich inphenolic compounds. Gokcen et al. [78] reported that the phenolic compounds widely found in grape berries are syringic acid, vanillic acid, gallic acid, *p*-coumaric acid, caffeic acid and ferulic acid. Gokturk Baydar et al. [53] reported that grape seeds richer than peels in terms of phenolic compounds including syringic acid, vanillic acid, gallic acid, *p*-coumaric acid, caffeic acid and ferulic acid. Phenolic compounds play a role in many physiological events such as color and taste formation in plants. 

## 4. Conclusions

The results obtained showed that the seeds of nine ‘Karaerik’ grape clones had higher total phenolic content and antioxidant activity values than peel and pulp. In addition, the total phenolic content and antioxidant activity was found clone dependent. Is is very important in grape breeding to use better clones in breeding activities in order to obtain nutraceutical rich ‘Karaerik’ plants. Moreover, the results indicated that the ‘Karaerik’ grape clones studied in this research may have great potential to be exploited by the grape processing industry. Further studies will be focused on the more detailed analysis in the different parts of these clones to clarify the beneficial effects on human health. In this context, the most promising clones—such as 7, 8 and 9— that have higher antioxidant activity may have present great potential for grape breeders, the food industry and health-conscious consumers. Clones obtained from the study could be used to establishing a base of vineyards where propagation can start to supply growers and propagators.

## Figures and Tables

**Table 1:** Some morphological traits of nine ‘Karaerik’ clones.

Locations	Clones	Harvest Period	Number of Seed	Cluster Form	Cluster Weight (g)	Berry Weight (g)	Berry Peel Color	Usage
Üzümlü	Clone 1	Mid-season	2–3	Winged cylindrical	460	5.86	Black	Table
Üzümlü	Clone 2	Late season	3–4	Winged cylindrical	505	6.13	Black	Table
Üzümlü	Clone 3	Late season	3–4	Winged cylindrical	587	6.90	Black	Table
Üzümlü	Clone 4	Late season	2–3	Winged conical	433	5.21	Black	Table
Üzümlü	Clone 5	Mid-late	2–3	Irregular winged conical	456	5.60	Black	Table
Üzümlü	Clone 6	Mid-late	2–3	Irregular winged conical	412	5.42	Black	Table
Üzümlü	Clone 7	Mid-season	2–3	Conical	390	5.03	Black	Table
Üzümlü	Clone 8	Mid-late	2–3	Conical	383	4.67	Black	Table
Üzümlü	Clone 9	Mid-season	1–2	Conical	346	4.59	Black	Table

Mid-season: 1 September–1 October; Mid-late: 10 September–10 October; Late season:15 September–15 October.

**Table 2:** Total phenolic content in berries of nine ‘Karaerik’ grape clones.

Clones	Total phenolic content (mg GAE/100 g FW)		
	Peel	Pulp	Seed
Clone 1	117cd	2.816ef	204e
Clone 2	129c	2.775f	218d
Clone 3	111d	2.904e	211de
Clone 4	121cd	3.112d	207e
Clone 5	138b	3.178d	222cd
Clone 6	126c	3.386c	245a
Clone 7	134b	3.514b	233bc
Clone 8	149a	3.490bc	229c
Clone 9	143ab	3.715a	238b

Different letters in the same column indicate significant differences (*p* < 0.05).

**Table 3:** DPPH assay results of ‘Karaerik’ grape clones.

Clones	DPPH (μmol Trolox/100 g FW)		
	Peel	Pulp	Seed
Clone 1	1152c	289bc	1510e
Clone 2	1186bc	303bc	1778c
Clone 3	1080d	276c	1687d
Clone 4	1125cd	282bc	1542e
Clone 5	1260ab	322ab	1744c
Clone 6	1242b	310b	1865b
Clone 7	1286ab	330ab	1851b
Clone 8	1340a	347a	1846b
Clone 9	1316ab	341ab	1918a

Different letters in the same column indicate significant differences (*p* < 0.05).

**Table 4:** FRAP assay results of ‘Karaerik’ grape clones.

Clones	FRAP (μmol Trolox/100 g FW)		
	Peel	Pulp	Seed
Clone 1	3620cd	82e	42600cd
Clone 2	3940bc	97cd	45260bc
Clone 3	3544d	77e	39880d
Clone 4	3870c	90cd	43700c
Clone 5	3986bc	104c	48670b
Clone 6	4235b	86d	48020b
Clone 7	4436ab	112b	49300ab
Clone 8	4127bc	128a	52460a
Clone 9	4610a	120ab	50640ab

Different letters in the same column indicate significant differences (*p* < 0.05).

**Table 5:** TEAC assay results of ‘Karaerik’ grape clones.

Clones	TEAC (μmol Trolox/100 g FW)		
	Peel	Pulp	Seed
Clone 1	322bc	56e	1490bc
Clone 2	348bc	66d	1530bc
Clone 3	310c	50f	1464c
Clone 4	340bc	60def	1510bc
Clone 5	382ab	70cd	1610b
Clone 6	360b	76c	1580bc
Clone 7	374ab	98a	1682ab
Clone 8	394ab	80bc	1702ab
Clone 9	414a	87b	1730a

Different letters in the same column indicate significant differences (*p* < 0.05).

**Table 6:** Correlation between the TPC and antioxidant capacities by DPPH, FRAP and TEAC.

Heading	TPC			DPPH			FRAP			TEAC		
	Peel	Pulp	Seed	Peel	Pulp	Seed	Peel	Pulp	Seed	Peel	Pulp	Seed
TPC	1.00	1.00	1.00									
DPPH	0.84 **	0.82 *	0.87 **	1.00	1.00	1.00						
FRAP	0.81 **	0.75 *	0.80 **	0.72 *	0.68 *	0.70 *	1.00	1.00	1.00			
TEAC	0.85 **	0.82 **	0.80 **	0.55 *	0.50 *	0.52 *	0.73 *	0.65 *	0.67 *	1.00	1.00	1.00

TPC: Total Phenol Content; *:(*p* < 0.05); **:(*p* < 0.01).

**Table 7:** Phenolic compounds in peel of ‘Karaerik’ clones (mg/100 g FW).

Clones	*p*-coumaric	Caffeic Acid	Syringic Acid	Gallic Acid	Ferulic Acid	Chlorogenic Acid	Quercetin	Myricetin	Vanillic Acid
Clone 1	5.2ab	25.0bc	57.6de	13.2bc	3.8^NS^	4.2^NS^	0.8^NS^	0.6^NS^	0.2^NS^
Clone 2	5.5ab	26.2bc	58.2de	14.7bc	1.9	2.4	1.2	0.4	0.3
Clone 3	4.2bc	23.8bc	59.6d	16.0b	2.0	2.6	0.9	0.5	0.3
Clone 4	4.9b	22.0c	51.1f	11.2bc	1.7	3.0	1.1	0.5	0.2
Clone 5	6.2ab	28.4ab	56.0e	10.4c	3.2	6.0	2.4	1.7	0.5
Clone 6	5.9ab	27.7b	54.3cd	16.8b	3.0	3.6	1.4	0.8	0.2
Clone 7	6.8ab	31.0ab	70.3b	14.3bc	3.8	4.0	1.7	0.9	0.5
Clone 8	7.9a	35.7a	73.6a	17.8ab	2.4	2.9	1.0	0.8	0.6
Clone 9	7.6ab	32.6ab	68.2c	19.1a	3.6	2.5	1.9	0.6	0.5

Different letters in the same column indicate significant differences (*p* < 0.05); NS: Non significant.

**Table 8:** Phenolic compounds in pulp of ‘Karaerik’ clones (mg/100 g FW).

Clones	*p*-coumaric acid	Caffeic Acid	Syringic Acid	Gallic Acid	Ferulic Acid	Chlorogenic Acid	Vanillic Acid	Quercetin	Myricetin
Clone 1	0.2^NS^	0.9^NS^	1.1^NS^	0.5^NS^	0.2^NS^	0.3^NS^	0.1^NS^	0.2^NS^	0.3^NS^
Clone 2	0.1	0.4	0.6	0.3	0.4	0.3	ND	ND	ND
Clone 3	0.3	0.6	0.8	0.3	ND	0.1	0.1	0.1	0.1
Clone 4	0.1	0.5	0.8	0.5	0.1	0.2	0.2	ND	ND
Clone 5	0.2	0.3	0.6	0.4	ND	ND	ND	0.1	0.1
Clone 6	0.3	0.6	1.4	0.5	0.1	0.1	0.1	0.2	ND
Clone 7	0.1	1.1	1.0	0.4	ND	0.5	ND	ND	0.1
Clone 8	0.4	0.7	1.2	0.4	0.2	ND	0.2	0.1	ND
Clone 9	0.4	1.0	1.0	0.6	0.3	ND	0.1	0.1	0.2

Different letters in the same column indicate significant differences (*p* < 0.05): NS: Non Significant; ND: Non Determined.

**Table 9:** Phenolic compounds in seed of ‘Karaerik’ clones (mg/100 g).

Clones	Catechin	Quercetin	Gallic Acid	Chlorogenic Acid	*p*-coumaric Acid	Caffeic acid	Syringic Acid	Myricetin
Clone 1	23.2d	60.4bc	97.3bc	8.6ab	1.4^NS^	2.3^NS^	1.7^NS^	1.9^NS^
Clone 2	26.6c	63.5b	102.2b	11.9a	1.0	2.8	1.1	1.4
Clone 3	20.6de	57.5c	104.9abc	10.2ab	2.1	1.9	2.2	2.0
Clone 4	19.5e	66.4ab	95.6c	4.1b	3.1	3.0	2.7	3.0
Clone 5	25.0cd	60.9bc	110.1a	7.8ab	4.2	1.9	2.0	1.5
Clone 6	31.1ab	70.4ab	104.0	9.8ab	4.0	3.7	4.4	2.9
Clone 7	29.4bc	65.0ab	99.2bc	6.8ab	2.7	4.5	4.5	ND
Clone 8	33.2a	72.1a	95.0c	4.0b	3.2	3.8	2.9	3.1
Clone 9	30.1b	63.3b	106.8	7.3ab	3.6	3.0	4.2	3.3

Different letters in the same column indicate significant differences (*p* < 0.05): NS: Non Significant; ND: Non Determined.

## Data Availability

All-new research data were presented in this contribution.
